# Presymptomatic Amyotrophic Lateral Sclerosis: From Early Biomarker Detection to Phenoconversion Prediction

**DOI:** 10.3390/diagnostics16172857

**Published:** 2026-09-05

**Authors:** Min Chen, Hexian Li, Qingwen Jin

**Affiliations:** Department of Neurology, Sir Run Run Hospital, Nanjing Medical University, No. 109 Longmian Avenue, Nanjing 211112, China; chenmin226@stu.njmu.edu.cn (M.C.); 15862610446@163.com (H.L.)

**Keywords:** amyotrophic lateral sclerosis, presymptomatic ALS, phenoconversion, neurofilament light chain, mild motor impairment, predictive biomarkers, genetic ALS, longitudinal monitoring

## Abstract

Amyotrophic lateral sclerosis (ALS) usually enters diagnostic pathways after motor symptoms emerge, by which time neural injury has been ongoing. Long-term hereditary ALS cohorts show that some pathogenic-variant carriers may show elevated neurofilament light chain (NfL), mild motor impairment (MMI), electromyographic abnormalities, or imaging changes before clinical manifestation. Since 2022, research has shifted from detecting presymptomatic abnormalities to identifying observable prodromal phenotypes and predicting phenoconversion timing. Operational MMI criteria, longitudinal imaging in chromosome 9 open reading frame 72 (C9orf72) expansion carriers, TAR DNA-binding protein 43 (TDP-43)-related fluid biomarkers, and plasma proteomic models spanning prediction horizons have broadened early identification. The ATLAS study, a trial of tofersen initiated in clinically presymptomatic carriers of superoxide dismutase 1 (SOD1) variants, incorporated specific SOD1 variants and within-person NfL increases into risk monitoring and used these criteria to select participants for the randomized treatment phase. Evidence remains concentrated in a few genetic subtypes, and no single marker accurately predicts individual phenoconversion. Identification requires genotype-specific natural history, serial clinical examinations and biomarker testing, with clinical utility validated in independent longitudinal cohorts and prevention trials.

## 1. Introduction

Amyotrophic lateral sclerosis (ALS) is a neurodegenerative disease characterized mainly by progressive degeneration of upper and lower motor neurons. Its clinical presentation and disease course are markedly heterogeneous [1]. ALS usually enters the diagnostic pathway after progressive limb weakness, bulbar dysfunction, or other motor symptoms emerge. Early manifestations and sites of onset vary widely, so patients may attend several consultations or initially receive other diagnoses. This often creates a substantial delay between symptom onset and definitive diagnosis [2]. By the time clinical features support a diagnosis, neurodegeneration has often been active for some time. To begin treatment before substantial functional loss, the window for identifying disease must move to the period before clinical manifestation. Long-term follow-up of hereditary ALS makes this type of research possible. Carriers of pathogenic variants in superoxide dismutase 1 (SOD1), chromosome 9 open reading frame 72 (C9orf72), or fused in sarcoma (FUS) can enter long-term follow-up before definite symptoms emerge. Researchers can follow the same individual across the continuum from an asymptomatic state through subtle abnormalities to clinically manifest disease [3,4]. However, a pathogenic variant establishes disease risk, not the timing of onset. Even carriers of the same variant may differ in age at onset, initial phenotype, and rate of disease progression. Presymptomatic research must go beyond detecting an abnormality. It must determine whether that abnormality reflects ongoing disease progression and whether it can estimate the risk of clinical manifestation over the next several months or years.

Longitudinal studies have documented several types of change before clinical manifestation. In some SOD1 pathogenic variant carriers, neurofilament light chain (NfL) levels in blood or cerebrospinal fluid rise before phenoconversion [3]. The Pre-Symptomatic Familial ALS Study (Pre-fALS) also found that mild motor impairment (MMI) and electromyographic evidence of denervation can precede clinically manifest ALS. The duration of this observable prodromal stage varies across genotypes [4]. In C9orf72 expansion carriers, longitudinal magnetic resonance imaging (MRI) identified specific atrophy patterns several years before symptoms in some phenoconverters. Other studies found changes in nerve excitability or cerebral metabolism [5,6,7]. In 2026, longitudinal plasma proteomics identified proteins that changed before phenoconversion and attempted to predict the risk of phenoconversion within 0.5–5 years [8]. This evidence shows that ALS-related changes can be detected before typical symptoms, but biomarkers differ in their timing, genotype applicability, and predictive ability. A single abnormal result is usually insufficient to establish near-term risk, whereas serial follow-up provides more informative within-person trajectories. This review examines how to identify high-risk populations and evaluates the strength of evidence for presymptomatic changes. It also considers phenoconversion prediction within defined time horizons and the implications of early identification for surveillance and preventive intervention.

### Literature Search and Evidence Selection

We conducted a targeted narrative search of PubMed for English-language, peer-reviewed reports relevant to human pathogenic-variant carriers or prediagnostic ALS published from 1 January 2000 through 30 August 2026. Search concepts covered presymptomatic or prodromal ALS, phenoconversion, MMI, SOD1, C9orf72, FUS, TAR DNA-binding protein gene (TARDBP), and relevant clinical, neurophysiological, imaging, and fluid biomarkers. Eligible reports addressed pathogenic-variant carriers or prediagnostic ALS, longitudinal natural history or biomarkers, presymptomatic or prevention trials, or clinical guidance for carrier follow-up. We gave highest priority to within-person longitudinal studies with observed phenoconversion. Longitudinal carrier studies without conversion were considered next, followed by cross-sectional studies when longitudinal evidence was unavailable. Studies in clinically manifest ALS were retained only when they informed biomarker specificity, disease-stage interpretation, or prevention-trial design. We excluded animal-only studies, purely mechanistic in vitro studies without direct relevance to human pathogenic-variant carriers, duplicate or superseded publications, and articles outside the review questions. Non-peer-reviewed sources were not used as scientific evidence. ClinicalTrials.gov was consulted only to verify the current recruitment, and follow-up status of ATLAS was not used as evidence of efficacy. The detailed Pre-fALS description of MMI as a prodromal state was published in 2022 [4], as was the ATLAS trial design for biomarker-guided tofersen treatment in clinically presymptomatic SOD1 carriers [9]. Because these two developments mark a transition toward clinically defined prodromal states and preventive intervention, Table 1 summarizes representative studies and trials published from 2022 onward. Earlier foundational studies that established relevant biomarker trajectories or clinical concepts remain integrated throughout the review.

## 2. Study Populations and Disease Trajectories in Presymptomatic ALS

### 2.1. SOD1 Variant Carriers Established the Basis for Presymptomatic Follow-Up

Natural history studies such as Pre-fALS recruit carriers without clinically manifest disease from families affected by ALS or ALS with frontotemporal dementia (ALS-FTD), together with non-carrier relatives from the same families. This design permits repeated neurological examinations, electromyography, and biospecimen collection in the same participant, so early changes can be related to subsequent clinical manifestation. SOD1 carriers were the earliest core study population because some variants have high penetrance and relatively consistent motor phenotypes and because SOD1 can be targeted directly. Cerebrospinal fluid SOD1 and blood NfL can reflect target lowering and the axonal injury response, respectively [10]. Early longitudinal evidence on NfL and the ATLAS tofersen trial in presymptomatic SOD1 variant carriers both arose from this framework [3,9].

Penetrance and the subsequent disease course differ markedly across SOD1 variants. In 2026, investigators examined exome data from approximately 470,000 UK Biobank participants. They identified 122 people carrying 19 pathogenic or likely pathogenic SOD1 coding variants, of whom 93.4% had no ALS record at the time of analysis. Another 535 participants were heterozygous carriers of the low-penetrance p.Asp91Ala variant [11]. Healthy volunteer bias, missed diagnoses in medical records, and follow-up duration may have influenced this proportion. Nevertheless, the findings show that detecting a SOD1 variant identifies a source of risk but cannot establish whether disease has progressed. Nor can it determine whether symptoms will emerge in the near term. Subsequent assessment must jointly consider the specific variant, the carrier’s age, age at onset among affected relatives, and serial within-person changes.

### 2.2. Different Genotypes Follow Distinct Trajectories Before Clinical Manifestation

In 2022, Pre-fALS reported 20 phenoconverters with hereditary ALS and provided a detailed view of genotype-specific clinical trajectories. Among 12 SOD1 A4V carriers, most developed sudden weakness, and only three had a brief motor prodrome lasting 1–3.5 months. By contrast, three SOD1 I113T carriers gradually developed diffuse upper and lower motor neuron signs over several years. Three C9orf72 repeat expansion carriers experienced focal or multifocal motor abnormalities for approximately 1–2 years [4]. The timing of NfL elevation likewise varied substantially by genotype. Levels usually rose 6–12 months before clinical manifestation in SOD1 A4V carriers. Changes were detected approximately 2 years beforehand in one FUS c.521del6 carrier and 3.5 years beforehand in one C9orf72 expansion carrier [12]. Few non-SOD1 phenoconverters have been studied in longitudinal cohorts. However, these individual observations suggest that NfL changes should not be interpreted using one shared time window across genotypes.

A C9orf72 repeat expansion may initially manifest as ALS, frontotemporal dementia (FTD), or a mixed phenotype. A UK Biobank study identified 701 expansion carriers among 490,331 participants. By age 80, the cumulative incidence of ALS or dementia was 66%, or 58% after accounting for competing mortality. Homozygosity for the unc-13 homolog A (UNC13A) risk allele was associated with higher risk [13]. Population data of this kind can estimate a carrier’s cumulative disease risk by a given age. However, they cannot predict whether an individual will first develop motor, cognitive, or behavioral changes. Longitudinal MRI has identified brain atrophy patterns several years before symptoms in some C9orf72 phenoconverters. Together, the population and imaging data indicate that monitoring should not focus only on the motor system [5].

### 2.3. Evidence from Presymptomatic Follow-Up of Other Genetic Subtypes and Sporadic ALS Remains Limited

Presymptomatic data remain limited for FUS, TARDBP, and other rare genetic subtypes. The FUS evidence comes mainly from individual phenoconverters and single-family observations, while TARDBP evidence includes a cross-sectional plasma microRNA signal in one family [4,12,14,15]. Abnormal aggregation of TAR DNA-binding protein 43 (TDP-43) is a shared pathological feature in most ALS, whereas classic SOD1- and FUS-associated ALS usually do not have TDP-43 aggregation as the predominant pathology [16]. This biological diversity makes genotype-specific natural history and serial sampling essential before monitoring rules can be transferred from one subtype to another.

Unlike hereditary ALS, sporadic ALS does not allow researchers to identify a high-risk carrier group in advance in the same way. Available early evidence comes mainly from prediagnostic biospecimens, longitudinal medical records, and retrospective symptom surveys conducted after disease onset. Nested case-control studies and the UK Biobank found that plasma NfL can rise before diagnosis in people who later develop ALS. In the UK Biobank, NfL exceeded the 95th percentile of age- and sex-matched controls approximately 2–3 years before diagnosis [17,18]. Simoni and colleagues also examined longitudinal medical records from 362 people later diagnosed with ALS and 1,448 matched controls. Creatinine, creatine kinase, body mass index, and several metabolic and hematological measures followed different group-level trajectories before diagnosis [19]. Van Wijk and colleagues found that some patients and caregivers recalled mild motor changes several months before typical weakness, bulbar symptoms, or respiratory symptoms [20]. These studies show that sporadic ALS can leave biological and clinical clues before diagnosis. However, none prospectively followed an asymptomatic general-population cohort from baseline. Such evidence cannot establish the duration of the clinically silent phase or directly identify people likely to develop ALS soon.

Because the detailed Pre-fALS description of MMI as a prodromal state and the ATLAS trial design were both published in 2022 [4,9]. Table 1 summarizes representative studies and trials from that year onward. Table 2 compares genotype-specific presymptomatic markers, observed lead times, and evidence strength.

**Table 1:** Representative Studies and Trials of Early Identification in Presymptomatic ALS since 2022.

Study	Population and Design	Main Findings	Contribution to Early Identification	Main Limitation
Benatar et al. (2022) [4]	20 phenoconverters with genetic ALS; prospective natural-history study	MMI preceded phenoconversion in 11 participants; trajectories differed among carriers of SOD1 A4V, SOD1 I113T, and C9orf72 repeat expansions	Proposed an observable motor prodrome	Few phenoconverters and uneven genotype distribution
Benatar et al. (2022) [9]	High-risk SOD1 variant carriers; ATLAS trial design	Specific variants and a within-person rise in NfL trigger entry into the randomized phase	Linked serial monitoring with a prevention trial	Entry criteria were derived mainly from rapidly progressive SOD1 variants
Dorst et al. (2023) [21]	Presymptomatic ALS gene carriers; body composition, resting metabolism, and NfL assessment	Body composition and resting metabolism differed between carriers and non-carriers when NfL did not differ significantly between groups	Extended presymptomatic evidence to systemic metabolic phenotypes	Limited sample size and genotype coverage
Irwin et al. (2024) [22]	Samples from genetically mediated ALS-FTD; fluid biomarkers of cryptic-exon-encoded proteins	A signal of lost TDP-43 function was detectable before clinical manifestation	Advanced molecular detection of pathology before clinical manifestation	Large-scale validation against time to phenoconversion is lacking
van Veenhuijzen et al. (2025) [5]	56 asymptomatic C9orf72 expansion carriers; longitudinal MRI	Five phenoconverters showed distinct atrophy patterns several years before symptoms	Linked imaging changes with observed phenoconversion	Only five participants phenoconverted
Benatar et al. (2025) [23]	31 phenoconverters, 153 carriers who had not phenoconverted, and 49 controls	Baseline MMI was associated with faster phenoconversion, with a hazard ratio of 5.1	Established operational MMI criteria and evidence for risk stratification	MMI was also common among carriers who had not phenoconverted
Gagliardi et al. (2026) [11]	Approximately 470,000 exomes from UK Biobank	Most carriers of pathogenic or likely pathogenic SOD1 variants had no ALS record	Demonstrated the importance of penetrance and the size of the carrier population	Healthy-volunteer and record-ascertainment biases
Ran et al. (2026) [8]	516 serial plasma samples; 33 phenoconverters	A 19-protein panel predicted phenoconversion over 0.5–5 years, with AUCs of 0.80–0.89	Moved the field from biomarker discovery toward prediction across multiple time horizons	Requires validation in an independent longitudinal cohort

Abbreviations: ALS, amyotrophic lateral sclerosis; ALS-FTD, amyotrophic lateral sclerosis with frontotemporal dementia; ATLAS, trial of tofersen in presymptomatic SOD1 variant carriers; AUC, area under the receiver operating characteristic curve; C9orf72, chromosome 9 open reading frame 72; MMI, mild motor impairment; MRI, magnetic resonance imaging; NfL, neurofilament light chain; SOD1, superoxide dismutase 1; A4V and I113T, SOD1 variants; TDP-43, TAR DNA-binding protein 43.

**Table 2:** Genotype-Specific Presymptomatic Markers, Observed Lead Times, and Evidence Strength.

Gene/Variant	Principal Presymptomatic Marker(s)	Observed Interval Before Phenoconversion	Current Evidence
SOD1 (A4V)	Serial serum, plasma, and CSF NfL and pNfH	Usually 6–12 months	Moderate–strong. Prospective cohorts included observed converters. ATLAS uses genotype plus serial NfL [3,9,12].
SOD1 (A4V, I113T, L106V)	MMI and EMG denervation MUNE Reduced or absent SICI	MMI: A4V, 1.2–3.6 months I113T, ≥1.6–7.5 years L106V, ≥4.4 years MUNE and SICI: several months	Moderate for MMI. Preliminary for MUNE and SICI because variant-specific samples are small [4,23,24,25].
C9orf72	Longitudinal MRI MMI and MBI	MRI: 4–68 months Motor MMI: approximately 11–12 months MBI with MMI: ≥21 months before bvFTD and ≥34 months before ALS-FTD	Moderate. Longitudinal studies included observed converters, although their number was small [4,5].
C9orf72	Serial NfL	≥4.7 years in one carrier	Moderate for detecting a presymptomatic rise. The quoted interval is based on one converter [12].
C9orf72	CSF UCHL1 and poly(GP) Cryptic-exon HDGFL2 Plasma microRNA SICI comparable with controls in one asymptomatic cohort	Not yet calibrated to phenoconversion	Preliminary. Carrier studies included few or no observed converters [22,26,27,28,29,30].
FUS (c.521del6)	Serial NfL and pNfH MMI and EMG abnormalities	Neurofilament elevation: ≥1.9 years MMI: 8.8 months	Preliminary. Timing is based on an individual converter [4,12].
FUS (P525L)	Cytoplasmic redistribution of FUS in fibroblasts	Approximately 2 years in one carrier	Preliminary. Single-family observation [14].
TARDBP (G376D)	Plasma microRNA dysregulation	Not established	Preliminary. Evidence is cross-sectional and family-specific. No prospective TARDBP converter series is available [15].

Lead times are observed intervals rather than validated individual prediction thresholds and may vary by variant, assay, and clinical endpoint. Evidence strength is a qualitative synthesis based on longitudinal follow-up, observed phenoconversion, replication, and sample size. TDP-43-related cryptic-exon and CSF seeding assays provide complementary proteinopathy evidence, but they have not established a conversion-calibrated interval in TARDBP carriers [22,31]. Abbreviations: A4V, I113T, and L106V, SOD1 variants; ALS-FTD, amyotrophic lateral sclerosis with frontotemporal dementia; ATLAS, trial of tofersen in presymptomatic SOD1 variant carriers; bvFTD, behavioral variant frontotemporal dementia; C9orf72, chromosome 9 open reading frame 72; CSF, cerebrospinal fluid; EMG, electromyography; FUS, fused in sarcoma; G376D, TARDBP variant; HDGFL2, hepatoma-derived growth factor-related protein 2; MBI, mild behavioral impairment; MMI, mild motor impairment; MRI, magnetic resonance imaging; MUNE, motor unit number estimation; NfL, neurofilament light chain; P525L and c.521del6, FUS variants; pNfH, phosphorylated neurofilament heavy chain; poly(GP), poly(glycine-proline); SICI, short-interval intracortical inhibition; SOD1, superoxide dismutase 1; TARDBP, TAR DNA-binding protein gene; UCHL1, ubiquitin carboxyl-terminal hydrolase isozyme L1.

## 3. Changes Before Clinical Manifestation and the Strength of Supporting Evidence

### 3.1. NfL: From Between-Group Differences to Longitudinal Monitoring Within Individuals

Serial sampling in Pre-fALS linked NfL changes to phenoconversion within the same individual. A 2018 study showed that serum and cerebrospinal fluid NfL abnormalities could appear approximately 1 year before clinical manifestation [3]. A 2019 extension found that NfL usually rose 6–12 months before clinical manifestation in SOD1 A4V carriers. The rise preceded manifestation by approximately 2 years in one FUS c.521del6 phenoconverter and by at least 3.5 years in one C9orf72 phenoconverter. Later follow-up extended the C9orf72 interval to at least 4.7 years [4,12]. Prediagnostic blood samples from people with sporadic ALS provided complementary evidence. Nested case-control studies and the UK Biobank both found that NfL rose before diagnosis [17,18]. A single archived sample can show that group-level differences precede diagnosis. Serial sampling reveals when an individual carrier’s NfL begins to rise, whether the increase persists, and whether it accelerates.

Comparisons across neurological disorders show why an elevated NfL value is not specific to ALS. In a 16-center single-molecule array (Simoa; Quanterix Corporation, Billerica, MA, USA) study, median serum NfL was 75.7 pg/mL (interquartile range [IQR], 48–123) in typical ALS, 45.1 pg/mL (26–81) in lower motor neuron-predominant ALS, 58.7 pg/mL (32–116) in upper motor neuron-predominant ALS, and 37.7 pg/mL (20–63) in primary lateral sclerosis [32]. Active peripheral axonal injury can enter the same range. In one serum electrochemiluminescence study, median NfL was 179 pg/mL in ALS, 123 pg/mL in Guillain–Barré syndrome, and 101 pg/mL in chronic inflammatory demyelinating polyneuropathy. The area under the receiver operating characteristic curve (AUC) for distinguishing ALS from the two inflammatory neuropathies was only 0.58 [33]. In multiple sclerosis, median serum NfL was 29.6 pg/mL (20.9–41.8) without gadolinium-enhancing lesions, 43.4 pg/mL (25.2–65.3) with enhancing lesions in either the brain or spinal cord, and 62.5 pg/mL (42.7–71.4) when both were involved [34]. Symptomatic genetic frontotemporal dementia had a median of 52 pg/mL (24–69) [35], while acute ischemic stroke reached a day-7 median peak of 211.2 pg/mL (104.7–442.6) [36].

Across these cohorts, NfL distributions overlapped substantially. Median values in Guillain–Barré syndrome, chronic inflammatory demyelinating polyneuropathy, and day-7 ischemic stroke were higher than the median in typical ALS, whereas values in active multiple sclerosis and symptomatic genetic frontotemporal dementia overlapped those reported for motor neuron-predominant ALS [32,33,34,35,36]. A single ALS-derived threshold could misclassify other forms of acute or chronic neuroaxonal injury as ALS. Numerical comparison also depends on specimen type and assay platform. Serum and ethylenediaminetetraacetic acid (EDTA) plasma concentrations differ systematically [37], and the cited cohorts used different Simoa and electrochemiluminescence configurations [32,33,34,35,36]. An isolated result should be interpreted against a matrix- and assay-matched reference distribution and in relation to age, renal function, body mass index, recent neurological events, and disease tempo [33,34,35,36,38]. During presymptomatic surveillance, sustained change from an individual baseline is more informative than a single cross-sectional cutoff. NfL indicates active neuroaxonal injury but does not by itself establish ALS.

After an NfL rise is attributed to ALS-related injury, its interpretation depends partly on where degeneration is active within the motor system. Median plasma NfL increased from 40.9 pg/mL in patients without clinically involved upper motor neuron regions to 97.2 pg/mL in those with involvement of three regions. This association remained significant after adjustment for age, sex, genetic status, baseline Revised Amyotrophic Lateral Sclerosis Functional Rating Scale (ALSFRS-R) score, progression rate, Medical Research Council (MRC) score, and King’s stage [39]. Senerchia et al. further showed that plasma NfL increased with upper motor neuron burden measured by the Penn Upper Motor Neuron Score and transcranial magnetic stimulation, independent of age, sex, progression rate, and phenotype. In the same study, plasma tau phosphorylated at threonine 181 (pTau181) was more closely associated with lower motor neuron involvement, particularly chronic denervation [40]. Earlier cohorts also related NfL to compound muscle action potential (CMAP)-defined lower motor neuron axonal loss and active spinal denervation [41,42]. Across this evidence, NfL appears to integrate axonal injury throughout the motor system, while recent plasma studies show a stronger association with upper motor neuron burden. Circulating NfL may reflect both disease tempo and the anatomical distribution of degeneration, beyond the clinical phenotype alone.

This distinction matters when NfL is followed before symptom onset. Similar plasma concentrations may arise from different combinations of cortical, corticospinal, bulbar, and lower motor unit injury. Serial NfL should be interpreted alongside upper motor neuron examination, transcranial magnetic stimulation (TMS), CMAP and needle electromyography, MMI assessment, and cognitive or behavioral evaluation. Genetic subtypes may differ in both disease tempo and the distribution of injury contributing to the circulating signal. These differences may alter the expected concentration and slope across SOD1, C9orf72, FUS, and TARDBP carriers. A meta-analysis of 60 studies and 8,801 participants supports the wider diagnostic and prognostic value of neurofilaments in ALS [43]. In presymptomatic surveillance, NfL is most informative as a sustained within-person trajectory interpreted within genotype-specific natural history and concurrent clinical or neurophysiological change.

### 3.2. MMI Makes the Motor Prodrome Clinically Observable

Prospective observation of 20 phenoconverters in Pre-fALS showed that 11 experienced MMI before developing clinically manifest ALS. The duration of this prodromal stage varied across genotypes [4]. The observable prodrome was usually brief in SOD1 A4V carriers. Motor abnormalities emerged more gradually over longer periods in SOD1 I113T and C9orf72 carriers. The study showed that subtle motor signs between ‘no abnormality’ and ‘clinically manifest ALS’ can be described as an intermediate clinical state. It also suggested that rapidly progressive variants may bypass a longer, observable prodromal phase.

Seven proposed research criteria for MMI were evaluated further in 2025. The study included 49 controls, 153 presymptomatic carriers who had not phenoconverted, and 31 phenoconverters. Before conversion, 17 of 31 phenoconverters (55%) had met MMI criteria. The criteria had also been met by 66 of 153 carriers who had not phenoconverted (43%) and 7 of 49 controls (14%). Baseline MMI was associated with a phenoconversion hazard ratio of 5.1 (95% confidence interval, 2.2–12.2). The times to 25% phenoconversion were 3.7 years with baseline MMI and 14.1 years without baseline MMI [23]. Both MMI detection and its association with phenoconversion weakened when electromyography was excluded. Together, MMI and electromyography identify a higher-risk motor prodrome in the Pre-fALS cohort. However, 43% of carriers who had not phenoconverted had also met the criteria. Inter-rater reliability and performance in independent, multicenter cohorts still require validation.

MMI remains a research-defined prodromal state rather than a formal ALS diagnosis. The Gold Coast criteria require progressive motor impairment together with defined upper and/or lower motor neuron dysfunction and exclusion of alternative disease processes [44].

### 3.3. Imaging, Neurophysiological, and Molecular Biomarkers Provide Complementary Evidence

Longitudinal imaging currently provides the evidence most closely linked to phenoconversion in C9orf72 carriers. A 2025 study followed 56 asymptomatic expansion carriers, 132 non-carrier relatives, and 359 population controls. During follow-up, five carriers phenoconverted to ALS or ALS-FTD. All five phenoconverters already showed specific cortical and subcortical atrophy patterns on their first presymptomatic scan. The earliest scan was obtained approximately 68 months before symptom onset [5]. Another study followed 66 presymptomatic C9orf72 carriers for approximately 3 years. It found longitudinal changes in the putamen, insula, and white matter measures, with some imaging changes associated with higher NfL levels [45]. The first study linked imaging to observed phenoconversion events, whereas the second described an average group trajectory. Together, they show that imaging can complement NfL by providing information about the affected regions and networks.

Peripheral nerve excitability, motor unit number estimation, ultrahigh-field magnetic resonance spectroscopy, and metabolic studies extend assessment beyond structural imaging. Small studies have reported presymptomatic changes in neural function, cerebral metabolism, and systemic metabolism [6,7,21]. A 2026 study also linked body-composition differences in C9orf72 carriers to cortical and subcortical measures, including thalamic volume [46]. Used serially, these methods may help localize early dysfunction and relate its spread to phenoconversion.

Threshold-tracking TMS provides a cortical counterpart to electromyography and peripheral nerve measures. In a longitudinal SOD1 study, short-interval intracortical inhibition (SICI) remained comparable with control values in 14 carriers who did not phenoconvert. In contrast, SICI was absent in two carriers who developed ALS within 3 months, while a third later converter showed a 32% reduction 8 months before weakness appeared [24]. This pattern suggests that loss of intracortical inhibition may signal proximity to phenoconversion rather than a stable feature of SOD1 carriage. The trajectory may differ by genotype. In 11 asymptomatic C9orf72 expansion carriers followed clinically for 3 years, SICI did not differ from control values at the study assessment. Symptomatic C9orf72-associated ALS, however, was associated with reduced SICI, increased intracortical facilitation, and larger motor-evoked potentials [29].

After symptom onset, cortical excitability changes with disease stage and the physiological measure used. Repeated threshold-tracking TMS showed further loss of SICI over 6–12 months in patients with recordable cortical responses [47]. The motor-evoked potential to compound muscle action potential (MEP:CMAP) ratio captures a wider range of corticospinal function, including hyperexcitable, hypoexcitable, and inexcitable states. In a 16-center cohort of 743 patients, higher ratios were more common in early disease and lower motor neuron-predominant phenotypes. Low or absent cortical responses occurred more often in upper motor neuron-predominant phenotypes and advanced King’s stages. A higher MEP:CMAP ratio independently predicted shorter survival, with a hazard ratio of 1.84 (95% confidence interval, 1.12–3.03) [48]. The available data support a stage-dependent progression from early disinhibition and hyperexcitability to reduced or absent corticospinal output. Serial TMS may complement NfL and electromyography in two settings: detecting emerging cortical dysfunction before phenoconversion and tracking corticospinal change after clinical manifestation. Prospective multicenter studies with serial measurements are needed to define these within-person transitions.

Other cerebrospinal fluid proteins may provide early biochemical information beyond that captured by NfL as a marker of axonal injury. Dellar and colleagues compared 48 asymptomatic C9orf72 expansion carriers with 39 non-carrier relatives. Cerebrospinal fluid ubiquitin carboxyl-terminal hydrolase isozyme L1 (UCHL1) was elevated in carriers, whereas NfL did not differ significantly between groups. The difference in UCHL1 remained after adjustment for age, sex, and NfL [26]. The UCHL1 result suggests that proteostasis-related changes may already exist in C9orf72 carriers before a between-group difference in NfL emerges. However, cross-sectional data cannot establish whether UCHL1 continues to rise as clinical manifestation approaches. Pathology-related fluid assays provide a separate form of molecular information. Poly(glycine-proline) [poly(GP)] can be detected in cerebrospinal fluid from C9orf72 expansion carriers before clinical manifestation. It marks protein products of the repeat expansion and may serve as a candidate pharmacodynamic biomarker for therapies targeting the repeat-expansion transcript. However, poly(GP) levels have not shown a consistent association with the timing of ALS or FTD manifestation [27,28]. A 2024 study measured a neoepitope of hepatoma-derived growth factor-related protein 2 (HDGFL2). The neoepitope is encoded by a cryptic exon after loss of TDP-43-mediated splicing repression. Related signals were detected in cerebrospinal fluid and blood from presymptomatic C9orf72 expansion carriers [22]. A 2025 study of TDP-43 seed amplification included only 14 presymptomatic participants with genetic ALS/FTD. Positive results and clinical phenoconversions during follow-up came mainly from carriers of granulin precursor (GRN) variants. Given its sample composition, this study is best viewed as a proof of concept for genetic TDP-43 proteinopathies. It cannot yet be extrapolated as a phenoconversion prediction tool for ALS in general [31]. Overall, NfL and MMI currently have the strongest links to observed phenoconversion. Longitudinal MRI adds information about the distribution of affected regions. UCHL1 suggests that biochemical changes may exist before NfL differs between groups. Poly(GP) and TDP-43-related assays remain more relevant to target confirmation and molecular subtyping.

Circulating microRNAs are small non-coding ribonucleic acids that can be measured in several biofluids and may reflect pathways involving TDP-43 and FUS. Genotype-defined family studies identified distinct plasma signatures in presymptomatic C9orf72 expansion carriers and in asymptomatic G376D TARDBP carriers [15,30]. These profiles provide candidate molecular markers for genotypes with limited longitudinal evidence. Serial sampling through observed phenoconversion is needed to determine whether the signals track disease activity or remain stable features of carrier status. A recent review summarizes the broader diagnostic, prognostic, staging, and treatment-monitoring evidence for microRNAs in ALS [49].

The principal evidence and current uses of these markers are summarized in Table 3.

## 4. From Early Changes to Phenoconversion Prediction

### 4.1. No Single Measure Can Yet Predict the Timing of Phenoconversion

NfL, MMI, and imaging abnormalities show that changes may already be under way in some carriers before clinical manifestation. However, evidence that a change has occurred does not mean phenoconversion will follow within a defined period. MMI is associated with faster phenoconversion, but it is also found in many carriers who have not yet converted. NfL provides temporal information, although its window of elevation varies with genotype and the rate of disease progression. Imaging abnormalities in C9orf72 carriers may emerge several years in advance. For practical prediction, the specific variant and family history establish the baseline risk. Age indicates where a carrier may lie within the natural history of the disease. MMI identifies an observable motor prodrome, while serial NfL measurements indicate the onset and acceleration of axonal injury. Together, these variables provide a reasonable basis for comparison. However, no externally validated model is currently applicable across all genotypes.

Swash and de Carvalho distinguished an undefined preclinical phase from the later transition to focal, progressive motor dysfunction [50]. The distinction supports serial monitoring because biological change may precede the clinically localizable transition that defines emerging disease.

### 4.2. Multiprotein Models Estimate Phenoconversion Risk Across Different Time Horizons

In 2026, Ran et al. examined whether plasma proteins could predict phenoconversion within the next 0.5, 1, 2, 3, or 5 years. Phenoconversion was defined as the onset of clinically manifest ALS and/or FTD in a presymptomatic carrier. The investigators analyzed 516 plasma samples. These included samples from 33 carriers who later phenoconverted and 10 high-risk carriers who did not phenoconvert during follow-up. Samples from 35 patients with clinically manifest ALS and 59 controls were also included. The investigators first compared patients with ALS and controls to identify disease-associated proteins. They then used serial preconversion samples from carriers to examine how these proteins changed over time. This process produced a 19-protein model that included NfL, reported under the analyte label NEFL in the proteomic dataset. For the 1-year prediction window, samples were assigned to the conversion group if clinically manifest ALS and/or FTD developed within the following year. Carriers who remained asymptomatic during that period served as the comparison group. The same approach was used for the other prediction windows. In fivefold cross-validation, the model achieved areas under the receiver operating characteristic curve (AUCs) of 0.80–0.89 across the 0.5-, 1-, 2-, 3-, and 5-year windows. The investigators also estimated time to clinical manifestation at preconversion visits among phenoconverters. The 19-protein panel had a mean absolute error of 1.62 years, compared with 2.37 years for NEFL alone [8]. This study advanced the field beyond comparisons between patients with ALS and healthy individuals. It attempted to identify which presymptomatic carriers might convert within defined periods and to estimate the approximate timing of clinical manifestation.

Using approved gene symbols, the core panel comprised NEFL, DUSP29, CALCA, NEB, NGRN, MYL11, NOS1, MYH1, TNNC1, DTNB, MEGF10, SYNM, APOA4, ART3, MYL3, EPHA1, EDA2R, TTN, and ACTN2. NfL appears under the source study’s analyte/gene-symbol label NEFL. The complete panel composition and end-to-end analysis scripts are publicly available in the source study’s GitHub repository. Participant-level discovery-cohort Olink (Olink Proteomics AB, Uppsala, Sweden) and phenotypic data are available through controlled request and a data transfer agreement, while UK Biobank data require approved access. The public repository does not include the participant-level data, horizon-specific fitted coefficients, intercepts, numerical thresholds, or trained model objects needed to run the published panel as a fixed individual-risk calculator [8]. The available resources specify the candidate panel and make the analytical workflow inspectable, but they do not yet permit direct application of the published prediction model.

The UK Biobank proteomic dataset included measurements for 15 of the 19 core proteins. MYL11, MYH1, TNNC1, and SYNM were unavailable. This 15-protein subset estimated time to clinical manifestation with a mean absolute error of 2.75 years, compared with 3.61 years for NEFL alone [8]. The analysis reproduced the direction of change for seven proteins and supports the added biological information of a multiprotein approach. Independent prospective validation will require a prespecified panel, fixed prediction equations, and serial samples from a separate carrier cohort.

## 5. Implications of Early Identification for Follow-Up and Preventive Intervention

### 5.1. Risk Stratification First Reshapes Follow-Up and Genetic Counseling

Multidisciplinary guidance published in 2025 recommended personalized follow-up for carriers of pathogenic ALS/FTD variants. Follow-up should consider genotype, age, family background, and individual preferences. Assessment should cover motor function, cognition and behavior, lifestyle, and reproductive choices. Routine disease-modifying treatment outside a clinical trial is not recommended while presymptomatic efficacy and long-term safety remain unproven [51]. At present, risk stratification is used mainly to plan research follow-up. This may include reviewing serial NfL changes, repeating neurological examinations, and discussing suitable clinical trials. Specialist teams should decide whether to add electromyography or cognitive and behavioral assessments. These decisions should reflect genotype, natural history, and newly emerging clinical changes. No uniform monitoring interval has been established across genotypes.

Genetic risk information can influence psychological well-being and major life decisions. In a survey of 90 asymptomatic people at risk of familial ALS, gene-positive respondents reported greater negativity, uncertainty, and psychological burden, whereas untested respondents thought about their risk more often and reported greater decisional regret [52]. A scoping review of 30 studies likewise found responses ranging from relief and greater preparedness to guilt, persistent worry, and marked distress [53]. Genetic status, prodromal abnormalities, and estimates of phenoconversion risk are related but distinct forms of information and should be discussed separately. A biomarker alert may prompt repeated blood sampling, electromyography, lumbar or intrathecal procedures, or trial entry while still leaving the timing of clinical manifestation uncertain.

Before testing or longitudinal surveillance begins, the return-of-results plan should define which findings will be disclosed, by whom, and in what setting. A pathogenic or likely pathogenic familial variant should be distinguished from an uninformative negative result and from a variant of uncertain significance. A variant of uncertain significance should not be treated as causal or used for predictive testing in relatives [54,55]. When exome or genome sequencing is used, consent should also distinguish actionable secondary findings that are deliberately sought from incidental findings encountered outside the primary diagnostic question. Current American College of Medical Genetics and Genomics (ACMG) guidance recommends pretest discussion and an opportunity to opt out of secondary findings. The current actionable-gene list is maintained separately [56,57]. These genomic findings are distinct from surveillance findings such as MMI, cognitive or behavioral change, electromyographic abnormalities, or a rise in NfL. Before longitudinal surveillance starts, the care team should ask which potential prodromal clinical and biomarker findings the carrier wishes to receive, obtain consent for their disclosure, and agree how findings documented in the medical record will be reviewed with the carrier [51].

Each returned result should be paired with its current clinical meaning and an agreed next step. For a pathogenic variant, counseling should address penetrance, uncertainty in age at onset, family communication, reproductive options, research and prevention trials, and jurisdiction-specific privacy and insurance implications. For a surveillance abnormality, the explanation should state how it changes planned follow-up or trial eligibility rather than presenting it as a fixed date of phenoconversion. Follow-up counseling should remain available after positive, negative, uncertain, secondary, or incidental results because emotional and family effects are not confined to a positive test. Disclosure preferences should be revisited as personal circumstances, variant classification, and biomarker evidence change [51,53,54,55,56,57].

### 5.2. ATLAS Translates NfL Monitoring into a Preventive Trial

The randomized trial of tofersen in symptomatic disease showed reductions in cerebrospinal fluid SOD1 protein and plasma NfL. These changes demonstrated target engagement and an effect on a marker of axonal injury. However, the primary clinical endpoint at 28 weeks did not reach statistical significance [10]. Open-label and extension data showed numerical long-term differences between earlier-start and delayed-start groups. A 2026 long-term analysis further reported outcomes with continued treatment [58]. These observations provide pharmacodynamic and exploratory long-term support for earlier intervention. However, they cannot replace a randomized test of whether presymptomatic treatment can delay clinical manifestation.

ATLAS (NCT04856982) was designed to create a pathway from natural history monitoring to preventive treatment. It enrolled clinically presymptomatic adults carrying highly or fully penetrant SOD1 variants associated with rapid progression. Plasma NfL was measured monthly during the natural history phase. Participants entered the randomized tofersen-versus-placebo phase only when NfL met two thresholds: at least 44 pg/mL and at least 10 pg/mL above the individual’s baseline. Other causes of NfL elevation also had to be excluded, and participants could not yet have developed clinically manifest disease [9]. The 44 pg/mL threshold came from trajectory modeling of Pre-fALS phenoconverters with rapidly progressive SOD1 variants. The derivation sample consisted mainly of A4V carriers, and the threshold depends on the specified assay platform. This threshold is an entry criterion for a particular trial, not a universal treatment threshold for all SOD1 carriers. According to the ClinicalTrials.gov update of 30 March 2026, ATLAS had enrolled 158 participants. Recruitment had stopped, follow-up was continuing, and primary endpoint assessment was expected in 2027. No presymptomatic efficacy results had been reported [59]. ATLAS currently provides a testable pathway for early intervention. Whether earlier tofersen treatment can delay clinical manifestation remains to be determined by the trial results.

### 5.3. Different Genotypes Require Distinct Intervention Pathways

C9orf72 carriers may develop ALS, FTD, or a mixed phenotype. Their NfL elevation is also less confined to a short window than in some rapidly progressing SOD1 variants. A 2025 study of prevention-trial design proposed considering motor, cognitive, and behavioral outcomes together [60]. A phase 1 trial of the C9orf72 antisense oligonucleotide BIIB078 in symptomatic disease did not support further development. The program was subsequently terminated [61]. This experience shows that a clearly defined genetic target does not automatically ensure an effective treatment. Dose, target biology, safety, and pharmacodynamic effects in humans must still be established step by step. For FUS, TARDBP, and other rare genotypes, genotype-specific natural-history data, serial biomarkers, and phenoconversion events must first be characterized. Primary outcomes and intervention windows can then be defined. Separate natural-history datasets, predictors, and intervention criteria may be needed for different genetic subtypes.

The applicability of current evidence to Chinese populations is another concern. A genetic analysis of 1,672 Chinese patients with ALS found pathogenic variants in 10.5%. The most common genes were SOD1, FUS, and annexin A11 (ANXA11) [62]. Current presymptomatic natural history data, NfL trigger criteria, and phenoconversion models come mainly from North American cohorts enriched for SOD1 A4V. They also draw on European and North American cohorts of C9orf72 carriers. The genetic composition of these populations differs from that of Chinese cohorts. Existing thresholds and risk models require new longitudinal validation in Chinese families and in variants common in China. Cross-sectional genetic profiles of affected patients alone are insufficient.

## 6. Conclusions

Early studies focused mainly on whether presymptomatic abnormalities existed. More recent longitudinal studies have begun to examine how these abnormalities relate to the timing of phenoconversion. MMI makes the motor prodrome observable in some carriers, while serial NfL places axonal injury on an individual timeline. Longitudinal imaging in C9orf72 carriers and pathology-related fluid assays add information about affected regions and molecular subtypes. In 2026, proteomic research also began to predict phenoconversion over periods ranging from several months to several years. The field has moved from asking whether an abnormality exists to asking how much near-term risk it signals once detected.

Observed phenoconversion events remain concentrated mainly among SOD1 and C9orf72 carriers. The temporal window and clinical meaning of a single measure vary by genotype, and multimarker models still lack independent longitudinal validation. ATLAS offers a representative pathway linking genotype, serial NfL monitoring, and preventive treatment. Whether this approach can truly delay clinical manifestation remains to be determined by the trial results. Future studies should harmonize clinical endpoints across larger, multicenter, long-term cohorts and validate individual-level risk estimates. They should assess predictive accuracy together with monitoring burden, genetic counseling, and the benefits of intervention. Digital speech measures could provide frequent, low-burden longitudinal data [63]. Their ability to detect presymptomatic change should be tested in genetically defined carrier cohorts. Clinical implementation will require evidence that defines whom to monitor, which serial measures to use, how to estimate near-term risk, and whether earlier intervention improves outcomes.

## Figures and Tables

**Table 3:** Principal Evidence and Current Uses of Markers in Presymptomatic ALS.

Marker	Strongest Current Evidence	Main Use	Question Requiring Validation
Serial blood or CSF NfL	Repeated sampling shows elevation before phenoconversion in some carriers [3,12]	Identify the onset and acceleration of axonal injury	Adjustment for age and kidney function, individual baselines, and conversion-time thresholds across genotypes and platforms
MMI and electromyography	Prospective phenoconversion cohorts and operational MMI criteria [4,23]	Identify a motor prodrome and support clinical risk stratification	Specificity, inter-rater reliability, and external multicenter validation
Longitudinal MRI	Presymptomatic scans showed distinct atrophy in five C9orf72 phenoconverters [5]; another cohort showed longitudinal group changes in the putamen, insula, and white matter [45]	Localize affected regions and complement assessment of frontotemporal system changes	More observed phenoconversion events and calibration of near-term risk
Peripheral and cortical neurophysiology; metabolism	MUNE, electromyography, and threshold-tracking TMS changed before phenoconversion in individual SOD1 carriers. C9orf72 nerve/cortical excitability and metabolic studies show genotype- and stage-related patterns [6,7,21,24,25,29,47,48]	Localize emerging cortical or motor-unit dysfunction and complement serial NfL	Serial within-person trajectories across genotypes and conversion-calibrated interpretation
poly(GP) and TDP-43-related assays	Genotype- or proteinopathy-related signals can be detected before clinical manifestation [22,27,28,31]	Confirm target presence, support molecular subtyping, and assess pharmacodynamic responses	A stable relationship with the timing of ALS or FTD phenoconversion
Multi-protein panels	Longitudinal proteomics predicted phenoconversion across several time horizons [8]	Integrate multisystem trajectories and estimate near-term risk	Full replication in an independent longitudinal carrier cohort

Abbreviations: ALS, amyotrophic lateral sclerosis; C9orf72, chromosome 9 open reading frame 72; CSF, cerebrospinal fluid; FTD, frontotemporal dementia; MMI, mild motor impairment; MRI, magnetic resonance imaging; MUNE, motor unit number estimation; NfL, neurofilament light chain; poly(GP), poly(glycine-proline); SOD1, superoxide dismutase 1; TDP-43, TAR DNA-binding protein 43; TMS, transcranial magnetic stimulation.

## Data Availability

No new data were created or analyzed in this study. Data sharing is not applicable to this article.
